# An appeal for strengthening genomic pathogen surveillance to improve pandemic preparedness and infection prevention: the German perspective

**DOI:** 10.1007/s15010-023-02040-9

**Published:** 2023-05-02

**Authors:** Bernd Salzberger, Alexander Mellmann, Anna Bludau, Sandra Ciesek, Victor Corman, Alexander Dilthey, Tjibbe Donker, Tim Eckmanns, Richard Egelkamp, Sören G. Gatermann, Hajo Grundmann, Georg Häcker, Martin Kaase, Berit Lange, Martin Mielke, Mathias W. Pletz, Torsten Semmler, Andrea Thürmer, Lothar H. Wieler, Thorsten Wolff, Andreas F. Widmer, Simone Scheithauer

**Affiliations:** 1 Department for Infection Control and Infectious Diseases, University Hospital Regensburg, Franz-Josef-Strauß-Allee 11, 93053 Regensburg, Germany; 2 Institute for Hygiene, University Hospital Münster, Robert-Koch-Straße 41, 48149 Münster, Germany; 3 Department for Infection Control and Infectious Diseases, University Medical Center (UMG), Georg-August University Göttingen, Robert-Koch-Straße 40, 37075 Göttingen, Germany; 4 Institute of Medical Virology, University Hospital Frankfurt, Goethe University, Frankfurt Am Main, Germany; 5 Institute of Virology, Charité Universitätsmedizin Berlin, Berlin, Germany; 6 Institute of Medical Microbiology and Hospital Hygiene, University Hospital Düsseldorf, Heinrich-Heine-University Düsseldorf, Düsseldorf, Germany; 7 Institute for Infection Prevention and Hospital Epidemiology, University Medical Center Freiburg, Freiburg, Germany; 8 Robert Koch Institute, Berlin, Germany; 9 Next Generation Sequencing, Public Health Agency of Lower Saxony, Hanover, Germany; 10 Department of Medical Microbiology, Ruhr University Bochum, Bochum, Germany; 11 Faculty of Medicine, Institute of Medical Microbiology and Hygiene, Medical Centre University of Freiburg, Freiburg, Germany; 12 Department of Epidemiology, Helmholtz Centre for Infection Research, Brunswick, Germany; 13 Institute of Infectious Diseases and Infection Control, University Hospital, Jena, Germany; 14 Division of Infectious Diseases and Hospital Epidemiology, University Hospital Basel, Basel, Switzerland

**Keywords:** Genomics, Surveillance, Pandemic, SARS-CoV-2, Epidemiology

## Abstract

The SARS-CoV-2 pandemic has highlighted the importance of viable infection surveillance and the relevant infrastructure. From a German perspective, an integral part of this infrastructure, genomic pathogen sequencing, was at best fragmentary and stretched to its limits due to the lack or inefficient use of equipment, human resources, data management and coordination. The experience in other countries has shown that the rate of sequenced positive samples and linkage of genomic and epidemiological data (person, place, time) represent important factors for a successful application of genomic pathogen surveillance. Planning, establishing and consistently supporting adequate structures for genomic pathogen surveillance will be crucial to identify and combat future pandemics as well as other challenges in infectious diseases such as multi-drug resistant bacteria and healthcare-associated infections. Therefore, the authors propose a multifaceted and coordinated process for the definition of procedural, legal and technical standards for comprehensive genomic pathogen surveillance in Germany, covering the areas of genomic sequencing, data collection and data linkage, as well as target pathogens. A comparative analysis of the structures established in Germany and in other countries is applied. This proposal aims to better tackle epi- and pandemics to come and take action from the “lessons learned” from the SARS-CoV-2 pandemic.

## Background

Genomic pathogen surveillance played a central role in the response to the SARS-CoV-2 pandemic [2, 12]. The recognition of the value of a powerful infrastructure for genomic pathogen surveillance may be one of the key lessons learned from the pandemic [11]. Genomic pathogen surveillance uses genomic data to determine the epidemiology of SARS-CoV-2 variants in a given region [12]. Combining genomic data with individual-level index and contact data (e.g. cluster/outbreak, contact persons, network, setting of contact, vaccination status, travel history, hospitalization or death data) has proven to be a powerful tool to determine transmissibility, high-risk settings and vaccine efficacy and to evaluate the effect of non-pharmaceutical interventions [1, 6, 9, 13, 14].

European countries differed substantially in their ability to employ genomic pathogen surveillance effectively for monitoring and managing the pandemic [4]. The World Health Organization (WHO) has formulated a global strategy for genomic surveillance of pathogens with pan- and epidemic potential to unite, inform and strengthen genomic surveillance efforts at regional, national and global levels [21]. Also under the theme of pandemic preparedness, the science academies of the G20 member states have emphasized the importance of efficient networks for genomic epidemiological surveillance and platforms for data collection and sharing [16]. A key challenge, however, consists in the effective implementation of such recommendations in the context of established national frameworks and approaches.

This paper will use best-practice examples from Denmark and the UK to define and propose key elements for a national genomic pathogen surveillance network in Germany.

## Genomic surveillance of SARS-CoV-2 in Germany

SARS-CoV-2 genome sequencing for surveillance purposes in Germany is performed in a decentralized manner by commercial and non-commercial laboratories, including clinical, academic and public health diagnostic virology and infection prevention laboratories as well as the National Consultant Laboratory for coronaviruses.

In 2021, the national system for genomic surveillance of SARS-CoV-2 in Germany was established by a federal directive (Coronavirus Surveillance Verordnung—CorSurV). Important provisions of the system include (1) a requirement that all SARS-CoV-2 sequences generated in Germany, including technical metadata and a unique identifier, be uploaded to a central repository provided by the national public health authority, the Robert Koch-Institute (RKI); (2) a mechanism for adjusting the proportion of sequenced SARS-CoV-2 viruses of cases incidence-dependent manner, implemented as a variable threshold on the number of sequenced cases eligible for reimbursement as a proportion of total cases ranging from 1% during high-incidence periods to 10% during low-incidence periods; (3) definitions of minimal performance and quality metric requirements for reimbursement, such as a turnaround time of ≤ 10 days and ≤ 5% undefined bases in sequenced SARS-CoV-2 genomes.

Sequences collected by the RKI are made publicly available and the transmitted unique identifiers enable linkage on a per-case basis between viral genome sequences and positive diagnostic test (since January 2021) and healthcare (beginning in January 2023) data at the RKI; utilization of the generated viral genome sequencing data at the level of local public health authorities, for example for the investigation of outbreaks or transmissions chains, however, remains fragmentary. In addition, quality metrics on turnaround times for laboratory and clinical data transmission over and above the defined minimal performance requirements, as well as an assessment of the extent to which the isolates selected for sequencing represent a random sample, have not been reported.

Various initiatives and institutions complement the federal system of SARS-CoV-2 surveillance in Germany. The Global Initiative on Sharing All Influenza Data (GISAID, https://gisaid.org/) platform promotes the rapid sharing of data initially from all influenza viruses and later SARS-CoV-2 and is partly funded by the German federal government.GISAID played an instrumental role in enabling the sharing of SARS-CoV-2 sequencing data from Germany prior to the establishment of the federal surveillance system, enabling the incorporation of these sequences in early studies of SARS-CoV-2 genomic epidemiology [2].

GenSurV (https://num-genomische-surveillance.de/), a collaborative project that has been initiated by the National University Medicine Research Network (NUM, funded by the Federal Ministry of Education and Research (BMBF, https://www.netzwerk-universitaetsmedizin.de/), aims at providing a platform for integrating genomic surveillance data sources and expertise that exist in a decentralized and quality-assured manner across Germany. Specific aims of GenSurV include the development and evaluation of sampling and scaling strategies, the development of a central data platform (CoGDat) for data integration, the development and provision of bioinformatic algorithms and modeling and forecasting approaches and phenotypic characterization capabilities. GenSurV integrates and comprises university hospitals, the RKI, reference centers and consulting laboratories and commercial diagnostic laboratories. In proof-of-concept studies, GenSurV and the associated MolTraX (Molecular surveillance and infection chain tracing for local public health authorities, https://num-genomische-surveillance.de/) project (NUM, BMBF) also played important roles in showing the feasibility and potential utility of high-intensity regional genomic surveillance regimes in a German context, and in demonstrating the effective utilization of SARS-CoV-2 sequencing data at the level of local public health authorities [10, 20]. GenSurV thus complements the federal system of genomic surveillance described above by broadening the available expertise for data generation, analysis and interpretation through an academic network. It provides a framework for data sharing, joint research and additional decision-making support for public policy and epidemic management. Thus, a combined, structured approach building on broad expertise distributed throughout Germany should be applied. A well-defined established expert panel with structures, networks, processes and regular training is necessary to better provide leadership during epi- and pandemics.

## Genomic surveillance of SARS-CoV-2 in Germany compared to the UK and Denmark

Denmark and the UK are two European countries that demonstrated high rates of SARS-CoV-2 case sequencing rates as well as effective implementations of genomic surveillance programs. Groups from both countries rapidly published data on viral spread and associations between variants, clinical severity and vaccine efficacy [5–8]. The key elements of these successful countries form the foundation for a German genomic surveillance system.

Denmark and the UK both were able to use already established and continuously funded infrastructures for genomic surveillance. Denmark has built an especially high laboratory capacity for PCR-testing and sequencing. The sequencing rate of positive sampling reached 85% in mid-2022. By February 2022, the UK sequenced 12.1% (2.3 million) of all positive samples, Denmark sequenced 83% (2.7 million) while Germany sequenced 3.1% (457,000). While this difference should not be interpreted as evidence of better management, it shows a higher degree of preparation for a pandemic situation in terms of necessary processes and structures. In the UK, institutions like the COVID-19 Genomics UK (COG-UK) consortium and the Scientific Advisory Group for Emergencies (SAGE) demonstrate the value of incorporating broad academic expertise both for rapidly implementing genomic surveillance at the beginning of a pandemic as well as for the ongoing interpretation of surveillance data and providing policy advice [18].

A key element for many applications of genomic epidemiology is the linkage of genomic data with a complementary test, healthcare (hospitalization, intensive care unit), vaccination and epidemiological (age, travel history, contacts) data. Both Denmark and the UK achieved a high degree of data linkage. Denmark uses unique personal identifiers in many epidemiologic projects, whereas a unified healthcare structure (NHS) facilitates data linkage in the UK [1, 6, 9, 13]. Both countries have historically a high standard of epidemiological research and a public health-oriented health care system.

In general, scalability is much easier to achieve using established technical, personnel and procedural infrastructures.

Supported by additional funding made available in response to the pandemic, Germany successfully leveraged existing structures at the RKI and within its academic system to implement a genomic surveillance effort. However, compared to the UK and Denmark, the German approach remained limited in important respects. First, the proportion of sequenced viruses in cases remained relatively low and the linkage between viral genomes and complementary clinical and epidemiological data of individual cases remained fragmentary. Second, a deep integration of the national genomic surveillance effort with the data sources (e.g., contact tracing and travel history data) available to local public health authorities was not generally achieved. Third, the potential contributions of experts at laboratories, including academia laboratories from more than 30 university hospitals were not integrated. Furthermore, significant open questions remain with respect to the major issue of metadata linkage and its legal basis in Germany as well as the cost effectiveness of the implemented system.

## Structural requirements for the successful establishment of national genomic surveillance programs

Based on the comparative analysis of Germany, the UK and Denmark, we conclude that a successful implementation of genomic pathogen surveillance requires a holistic approach that extends and respects established national structures while learning from other countries and adopting international best practices to national requirements.

Structurally, this should involve (1) building on structures already established or initiated, (2) improving integration, collaboration and knowledge exchange between relevant institutions and stakeholders, (3) ensuring interoperability (to be able to ensure linkage with other national or international data sets), (4) ensuring the integration of metadata, e.g. through explicit legal provisions for record linkage and data sharing for the purposes of genomic surveillance, and (5) leveraging the capabilities of local public health authorities. In parallel, target pathogens for genomic surveillance should be identified in a collaborative manner, taking into account future pandemic potential as well as healthcare burden, e.g. in the case of multi-drug resistant bacteria. Further relevant issues include the leveraging of experts from all areas and institutions, including laboratory networks as well as defining uniform quality criteria and data-sharing standards.

## Recommendations for establishing a national network for genomic surveillance of pathogens beyond SARS-CoV-2 in Germany

Starting from the WHO strategy for genomic surveillance of pathogens with pan- and epidemic potential [21] and from the results of our comparative analysis to propose adapted recommendations for Germany with the goal of establishing a network for genomic pathogen surveillance, we recommend the following points:

### Stakeholders

A national network of genomic surveillance should be established in a joint effort of the RKI, public health authorities, universities, university hospitals, reference centers, consulting laboratories, scientific research centers, and commercial diagnostic laboratories. The selection of these stakeholders is based on their expertise and existing scientific or public health collaborations.

### Sequencing rates and sample selection

A sufficient sequencing rate of pathogen isolates is an important element of successful systems of genomic surveillance. Data of 1.2 million complete SARS-CoV-2 genome had been collected in Germany by the end of 2022. This illustrates the principal capacity that can be called upon in specifically created circumstances (legal, financial, pandemic situation). The national network for pathogen surveillance in Germany should thus develop strategies for achieving sufficient sequencing rates of relevant pathogens while maintaining or even improving cost efficiency. Experiences from the UK and Denmark show that increased funding in Germany may be required to achieve sufficient sequencing rates; however, in addition to the proportion of sequenced samples, the implemented sample selection strategies also play a key role in strengthening the utility of generated pathogen sequencing data for public health purposes. The most appropriate sampling strategy and frequency has to be defined for each pathogen, which may include randomized as well as event-driven sampling strategies (applicable, for example, in the context of suspected outbreaks or infection clusters). Sampling strategies may also incorporate factors like target population, location and seasonality.

### Selection of target pathogens

SARS-CoV-2 is only one use case; genomic surveillance has been successfully applied to bacterial outbreaks, e.g. in a nationwide outbreak of Shiga-toxin-producing *Escherichia coli* [15, 19]. The WHO has also stressed the role of genomic pathogen surveillance for infectious agents with pan- or epidemic potential. A well-considered, coordinated prioritization strategy based on scientific evidence and contributed by experts from all partners is crucial. Criteria informing this decision and selection should include: associated morbidity and mortality, the burden of disease and relevance for population health, and potential pressure on hospitals and the health care system as a whole. In addition to SARS-CoV-2, the list of pathogens that should be covered by genomic pathogen surveillance includes for example human and avian influenza viruses, Enterobacterales with antibiotic resistance to carbapenems and respiratory syncytial virus (RSV). The pandemic potential of influenza viruses is obvious, as is the role of Carbapenemase-producing Enterobacterales. RSV may well be an important use case for setting up genomic surveillance as new vaccines await their introduction [3].

### Clinical and epidemiological data linkage

The ability to link pathogen genome sequences with clinical and epidemiological records on a per-case level has been a key success factor of the genomic surveillance programs of the UK and Denmark. The linking of genomic to epidemiological data in Germany is a surprising but very substantial problem for academic research as much as for public health. Although the RKI has built up the capacity for linking test data (including sequence data) to hospitalization and mortality, this tool is currently only available for SARS-CoV-2. The inclusion of other pathogens will require legal enactment and can currently probably only be legally justified by public health concerns, not by academic interest. In contrast to Denmark and the UK, detailed clinical data from health care providers are not readily accessible in a unique repository due to hurdled concerns of privacy and data security. The national network for pathogen surveillance in Germany should contribute to the establishment of an explicit legal framework for the collection, sharing and analysis of pathogen genome sequencing data and metadata for the purposes of genomic pathogen surveillance by the German federal government.

### Technical standards, data formats and interoperability

The national network for pathogen surveillance in Germany should define appropriate technical standards and quality metrics for the selected target pathogens, providing, for example, recommended protocols for genome sequencing or assembly, incorporating established and emerging sequencing technologies. Existing international standards should be used whenever defined (e.g. GISAID or PUBMLST). The network should also contribute to the definition of data formats and standards that ensure interoperability with the datasets generated by other key projects and initiatives in the German clinical informatics context, such as the Medical Informatics Initiative Germany (MII) and other projects of the NUM.

### Institutional integration and collaboration

The national network of genomic surveillance in Germany should leverage existing initiatives like GenSurV, which play an important role in providing a framework for institutional integration, collaboration, and joint research and infrastructure development. Components of the GenSurV infrastructure, such as the CoGDat data hub, should be upgraded to support the target pathogens selected by the national network of genomic surveillance in Germany; a deeper integration of German local public health authorities into genomic surveillance activities should be supported by the MolTraX extension project of GenSurV.

## Next steps

We propose to build a collaborative German genomic pathogen surveillance network in a multifaceted process including the identification of pathogens to be targeted, setting up technical standards for sequencing and data collection, and defining technical and legal standards and procedures for the safe use of linked data [17]. The strategy should include pathogens that are of continuous high relevance, pathogens of high pandemic risk and other pathogens that are relevant in a variety of ways; prioritization of pathogens for genomic pathogen surveillance will be a key issue. Stakeholders in this process include public health organizations, universities, university and other large hospitals, reference centers, additional academic institutions and health care providers. To meet international standards and to enhance cooperation, we propose to either base reimbursement for genome sequencing on high and verifiable quality standards or to set up adequate funding for academic and involved laboratories within defined research studies or public health surveillance activities.

The network proposed here should be initiated and coordinated in a collaborative fashion regarding data analysis, data sharing and integration into international consortia. The various existing efforts (e.g. RKI; GenSurV, NUM) in Germany have to be brought together into one collaborative, interactive structure to benefit from each other's expertise and not at least to dissolve inefficient duplicate structures. Utilization of surveillance data obtained in such a network will benefit decision-makers in the scientific community, public health institutions and government.

## Conclusion

Establishing a national genomic pathogen surveillance network that links academic and other scientific centers, public health institutes, and diagnostic laboratories in Germany is a key priority to meet pandemic preparedness. An important prerequisite for realizing the full potential of pathogen genomic surveillance in Germany is the collaborative collection and sharing of sequencing data as well as the establishment of standards and mechanisms for the linkage between sequencing and epidemiological and clinical data. The national genomic pathogen surveillance network outlined above will address these requirements and benefit decision-makers in government, public health institutions and the scientific community. In the face of current potentially new epidemic threats, the protection of population health in Germany will heavily rely on such a functioning genomic pathogen surveillance network.

## Data Availability

Not applicable.
